# Mitochondrial ROS in *Slc4a11* KO Corneal Endothelial Cells Lead to ER Stress

**DOI:** 10.3389/fcell.2022.878395

**Published:** 2022-04-26

**Authors:** Rajalekshmy Shyam, Diego G. Ogando, Joseph A. Bonanno

**Affiliations:** Vision Science Program, School of Optometry, Indiana University, Bloomington, IN, United States

**Keywords:** corneal endothelial cells, ROS—reactive oxygen species, er stress, ERAD (ER associated protein degradation), MitoQ, SLC4A11 ammonia transporter

## Abstract

Recent studies from *Slc4a11* ^*−/−*^ mice have identified glutamine-induced mitochondrial dysfunction as a significant contributor toward oxidative stress, impaired lysosomal function, aberrant autophagy, and cell death in this Congenital Hereditary Endothelial Dystrophy (CHED) model. Because lysosomes are derived from endoplasmic reticulum (ER)—Golgi, we asked whether ER function is affected by mitochondrial ROS in *Slc4a11* KO corneal endothelial cells. In mouse *Slc4a11* ^*−/−*^ corneal endothelial tissue, we observed the presence of dilated ER and elevated expression of ER stress markers BIP and CHOP. *Slc4a11* KO mouse corneal endothelial cells incubated with glutamine showed increased aggresome formation, BIP and GADD153, as well as reduced ER Ca^2+^ release as compared to WT. Induction of mitoROS by ETC inhibition also led to ER stress in WT cells. Treatment with the mitochondrial ROS quencher MitoQ, restored ER Ca^2+^ release and relieved ER stress markers in *Slc4a11* KO cells *in vitro*. Systemic MitoQ also reduced BIP expression in *Slc4a11* KO endothelium. We conclude that mitochondrial ROS can induce ER stress in corneal endothelial cells.

## Introduction

Congenital Hereditary Endothelial Dystrophy (CHED) is a rare recessive blinding disease that affects 3 out of 100,000 newborns each year. In this disease, the corneal endothelial layer malfunctions resulting in corneal edema (Aldave et al., 2007). This progressive disease arises during infancy and has no cure, and the current treatment involves corneal transplantation (Aldave et al., 2013). Loss of function of a membrane protein, SLC4A11, leads to CHED (Vithana et al., 2006; Han et al., 2013). Recent studies from our lab and others indicate that in the absence of *Slc4a11*, glutamine-induced mitochondrial Reactive Oxygen Species (ROS) production results in lysosomal dysfunction, autophagy impairment, and aberrant master regulator of oxidative stress response Nrf2 signaling resulting in cell death (Guha et al., 2017; Ogando et al., 2019; Shyam et al., 2021).

In eukaryotes, lysosomes are formed from the budding off of acidic proteases from the ER-Golgi network. Since *Slc4a11* KO lysosomes are dysfunctional, we asked if there was Endoplasmic Reticulum (ER) stress as well. ER is the primary organelle in which protein biosynthesis, folding, and modifications occur. ER can also identify misfolded proteins and deploy them for ER-associated degradation (ERAD). The canonical ERAD system is characterized by the proteasome-mediated degradation of misfolded proteins, whereas autophagy is activated in the non-canonical mode (Ren et al., 2021). When the rate of misfolded protein formation saturates ERAD, a signaling cascade known as Unfolded Protein Response (UPR) is triggered leading to ER stress (Ren et al., 2021). In Fuchs Endothelial Corneal Dystrophy, ER stress (Jun et al., 2012) and activation of the unfolded protein response (Okumura et al., 2017b) are present. In addition, some mutations in *Slc4a11* that are associated with CHED and FECD results in ER retention of the misfolded protein (Loganathan and Casey, 2014). The protein folding process in the ER is affected by several internal and external cues, including [Ca^2+^] and oxidative stress (Xu et al., 2012). Since elevated mitochondrial oxidative stress (Guha et al., 2017; Ogando et al., 2019) and dysfunctional lysosomes are present in the CHED mouse endothelium (Shyam et al., 2021), we asked whether ER function is affected in this disease model.

In *Slc4a11* ^*−/−*^ corneal endothelium, we observed alterations in ER morphology and elevated expression of UPR associated proteins. In addition, we found that glutamine-induced mitochondrial ROS is the cause of ER stress in *Slc4a11* ^*−/−*^ corneal endothelial cells.

## Materials and Methods

### Animal Model

*Slc4a11* ^*−/−*^ (KO) mice were originally provided by Dr. Eranga Vithana (Singapore Eye Research Institute) (Vithana et al., 2006). *Slc4a11* ^*+/+*^ (Normal, Wild Type, WT) and KO mice were housed and maintained in pathogen-free conditions. Animals were used in the experiments in accordance with institutional guidelines and the current regulations of the National Institutes of Health, the United States Department of Health and Human Services, the United States Department of Agriculture and Association for Research in Vision and Ophthalmology (ARVO) Statement for the Use of Animals in Ophthalmic and Vision Research.

### Cell Culture Experiments

Conditionally immortalized mouse corneal endothelial cells (MCEC) *Slc4a11* ^+/+^ and *Slc4a11* ^−/−^ were generated and maintained in our lab (Zhang et al., 2017b). Cells were cultured in Complete Media, which contains OptiMEM-I medium (#51985; Thermo Fisher Scientific, Canoga Park, CA, United States), 14 mM Glucose and 4 mM L-Alanyl Glutamine supplemented with 8% heat-inactivated fetal bovine serum (FBS) (#10082139; Thermo Fisher Scientific), EGF 5 ng/ml (#01–107 Millipore, Darmstadt, Germany), pituitary extract 100 μg/ml (Hyclone 15 Laboratories, Logan, UT, United States), calcium chloride 200 mg/L, 0.08% chondroitin sulfate (#G6737; SigmaAldrich Corp., St. Louis, MO, United States), gentamicin 50 μg/ml (#15710072; Thermo Fisher Scientific), antibiotic/antimycotic solution diluted 1:100 (#15240062; Thermo Fisher Scientific), and 44 units/mL IFN-γ (#485-MI; R&D Systems, Minneapolis, MN, United States).

For experiments, cells were incubated in Assay Media, which contained Dulbecco’s Modified Eagle Medium (no glutamine, no sodium pyruvate, no phenol red, contains 5.5 mM glucose) (#11054001; Thermo Fisher Scientific), supplemented with 0.5 mM glutamine (#250030-081, Thermo Fisher Scientific) and 0.5% dialyzed FBS (#26400-036; Thermo Fisher Scientific) at 33°C for 16 h. Corneal endothelium is exposed to 0.5 mM glutamine *in vivo* (Langford et al., 2007), therefore this concentration was used in our experiments. Drug treatments, 2 μM MitoQ (#317102, Medkoo Biosciences, Morrisville, NC, United States), or 0.1 μM Thapsigargin (SML 1845, Sigma Aldrich) were added into the assay media for 16 h. All drug treatments were carried out in assay media. Significant elevation in mitochondrial ROS was observed after 16 h treatment of cells in assay media (Shyam et al., 2021).

### Real Time PCR

Total RNA was isolated using RNA mini kit (#74104, Qiagen, Germantown, Maryland, United States). 1μg of RNA was used to prepare cDNA using a high capacity RNA to DNA kit (#4388950, Thermo Fisher Scientific). Previously published primer designs were used to amplify XBP1, us-XBP1, s-XBP1, and β-actin (Yoon et al., 2019). NCBI primer designing tool was used to design all other primers used for this study. List of primers used can be found in Table 1. Real time PCR was conducted using SYBR green dye using a BioRad CFX96 system. Relative quantitation was performed using 2^−ΔΔCt^ method against housekeeping gene. Fold Change (FC) is calculated as 2^−∆∆CT^. Data is plotted on Log10 scale.

**TABLE 1:** Primer sequences used in this study.


m-sXBP1-F	CTG​AGT​CCG​AAT​CAG​GTG​CAG
m-sXBP1-R	GTC​CAT​GGG​AAG​ATG​TTC​TGG
m-usXBP1-F	CAG​CAC​TCA​GAC​TAT​GTG​CA
m-usXBP1-R	GTC​CAT​GGG​AAG​ATG​TTC​TGG
m-Total XBP1-F	TGG​CCG​GGT​CTG​CTG​AGT​CCG
m-Total XBP1-R	GTC​CAT​GGG​AAG​ATG​TTC​TGG

### Cytosolic Calcium Measurement

Immortalized *Slc4a11* ^+/+^ (WT) and *Slc4a11* ^−/−^ (KO) corneal endothelial cells were cultured on fibronectin pre-coated 25-mm diameter glass coverslips (GG-25-pdl; Neuvitro Corporation, Vancouver, WA, United States) for 24 h in Assay media with or without glutamine. 1 mM stock of Fura 2-AM (#F1221, ThermoFisher Scientific) was prepared in DMSO. 5 μL of Fura stock solution along with 5 μL of 20% Pluronic F-127 solution in DMSO (#P3000MP, ThermoFisher Scientific) were mixed in 1 ml Hanks Balanced Salt Solution (HBSS) to obtain a final Fura 2-AM concentration of 5 μM. Cells were loaded with Fura-2AM for 30 min at 37°C. Cover slips were washed in HBSS for 30 min at room temperature, before they were mounted into a perfusion chamber, connected to a stage warmer (37°C) of an inverted microscope (Eclipse TE200; Nikon, Tokyo, Japan). Cells were perfused with HBSS containing 500 μM non-cell permeant BAPTA for 50 s to establish baseline. After establishing the fluorescence baseline, cells were perfused with HBSS containing 50 μM Ionomycin (#2092, Tocris, Minneapolis, MN, United States). Perfusing solutions were adjusted to pH 7.5 using NaOH, and kept at 37°C in a warming box. The flow of the perfusate (∼0.5 ml/min) was by gravity. Osmolarity of all solutions was adjusted to 295 mOsm with sucrose. Cells were imaged with a 40x oil-immersion objective (Nikon). Cells loaded with Fura-2AM were excited at 340 and 380 nm and the emission was collected at 505 nm to measure changes in cytosolic calcium. Ca^2+^ bound Fura-2 has an excitation maximum of 340 nm, while Ca^2+^ free Fura-2 has its excitation maximum of 380 nm. In both states, the emission maximum is 510 nm. The 340/380 nm excitation ratio for Fura-2 is a measure of intracellular [Ca^2+^].

### Electron Microscopy

Endothelium-Descemet’s tissue samples from WT and KO mice were fixed with 2.5% glutaraldehyde (#16020, Electron Microscopy Sciences, Hatfield, PA, United States), 4% paraformaldehyde (#15710, Electron Microscopy Sciences), in 0.1 M sodium cacodylate buffer, pH 7.2 at 4°C and post-fixed with 1% osmium tetroxide (# 19,150, Electron Microscopy Sciences) in 0.1 M sodium cacodylate buffer (#12300, Electron Microscopy Sciences), pH 7.2 at 4°C. Samples were dehydrated in a graded ethanol series to 100% ethanol, transitioned to propylene oxide (#20401, Electron Microscopy Sciences), and infiltrated with Embed 812 resin (#14120, Electron Microscopy Sciences). Infiltrated samples were placed in flat embedding molds and polymerized at 65°C for 18 h. Resin blocks were cut with a diamond knife using a Leica Ultracut UCT ultramicrotome (Leica Systems, Buffalo Grove, IL). Sections were placed on 300 mesh copper TEM grids (#0300-CU, Electron Microscopy Sciences) and stained with saturated uranyl acetate in aqueous solution, and lead citrate. Stained sections were viewed with a JEM-1010 TEM (JEOL, Peabody, MA, United States) at 80 kV and photographed with a Gatan MegaScan 794 CCD camera or JEM-1400plus TEM (JEOL) with a Gatan OneView CMOS digital camera.

### Protein Simple—Simple Western Wes Immunoassay for Protein Expression

Corneal endothelial cell layer was removed from dissected corneas. Protein lysates were prepared by pooling the tissues from two animals, using radioimmunoprecipitation (RIPA) lysis buffer containing protease and phosphatase inhibitors. Equal amounts of protein (1.5 µg) were loaded into 12–230 kDa separation module kit, and analyzed using the Protein Simple Wes System (Protein Simple, San Jose, CA, United States) following the manufacturer’s instructions as previously described (Shyam et al., 2021). Antibodies used are BIP (#3177, Cell Signaling technologies, Danvers, MA, United States), GADD153 (#NB600-1,335, Novus, Centennial, CO, United States), and α-tubulin (#NB100-690, Novus).

Wes immunoassay was carried out since it is challenging to obtain sufficient total protein from corneal endothelial peelings to conduct traditional western blots. Our lab has used this approach in several recent studies to quantify changes in protein expression (Ogando et al., 2021; Shyam et al., 2021; Shyam et al., 2022; Ogando and Bonanno, 2022).

### Mitochondrial ROS Induction

*Slc4a11* ^+/+^ corneal endothelial cells were treated with 0.25 μM Antimycin A (#A8674, Sigma-Aldrich), 0.5 μM Rotenone (#R8875, Sigma-Aldrich), or 0.25 μM Antimycin A+ Rotenone in assay media for 18 h. Control cells were cultured in assay media for the same duration.

### Flow Cytometry for Mitochondrial ROS and Apoptosis

*Slc4a11* ^+/+^ corneal endothelial cells were trypsinized and stained in triplicate with Pacific Blue Annexin V kit with PI (#640928, BioLegend, San Diego, CA, United States) or MitoSOX (#M36008, Thermo Fisher Scientific) following manufacturer’s instructions. Cells were collected in 2 ml micro centrifuge tubes following filtration using CellTrics Fliters (#04-004-2,327, Sysmex, Gorlitz, Germany). Flow cytometry analysis was conducted on MACSQuant VYB (Miltenyi Biotech, Germany). 10,000 cells were collected for each acquisition. Data were analyzed using FCS Express (De Novo software, Pasadena, CA, United States).

### Aggresome Quantification

MCEC cultures in 12-well format were trypsinized, washed and fixed using 4% Paraformaldehyde. Following permeabilization, cells were stained for aggresomes using the Aggresome detection kit (#ENZ51035, Enzo Life Sciences, NY) following manufacturer’s instructions. The aggresome detection reagent emits strong fluorescence when bound by misfolded proteins but not in solution. Therefore, the increase in fluorescence intensity is indicative of the levels of protein aggregates present in the cells. After filtration using 50 μm sterile CellTrics Filters flow cytometry analysis was conducted on MACSQuant VYB. 10,000 cells were collected per acquisition. Cells treated with ER stress inducer, Thapsigargin (#1138, Tocris, Minneapolis, MN, United States), served as a positive control, unstained cells were used as negative control. Data were analyzed with FCS Express.

### MitoQ Injections

MitoQ, 1 mM (#317102; Medkoo Bisociences) was prepared in equal volume of ethanol: distilled sterile water mixture. 100 μL of this solution containing 68 μg of MitoQ was injected intraperitoneally into *Slc4a11* ^−/−^ mice on alternate days. Littermate control mice were injected with equal volume of vehicle for the same duration at the same time/day. To prevent stress induced weight loss due to increased handling, animals were provided with a vet-approved supplement. One tablespoon of commercially available peanut butter was provided to both control animals and experimental animals, once a day immediately after handling.

### Statistical Analysis

All experiments were performed at least three times on different days. Error bars represent mean ± SD. Statistical significance was calculated using unpaired t-tests when two groups were involved. ANOVA with Tukey’s multiple comparisons test was used to determine statistical significance if more than two groups were analyzed. Statistical analyses were conducted using Graph Pad Prism software (La Jolla, CA, United States).

### Model Figures

The model figures were created with the aid of BioRender.com.

## Results

### Activation of Unfolded Protein Response in *Slc4a11* ^*−/−*^ Corneal Endothelium

**FIGURE 1:**
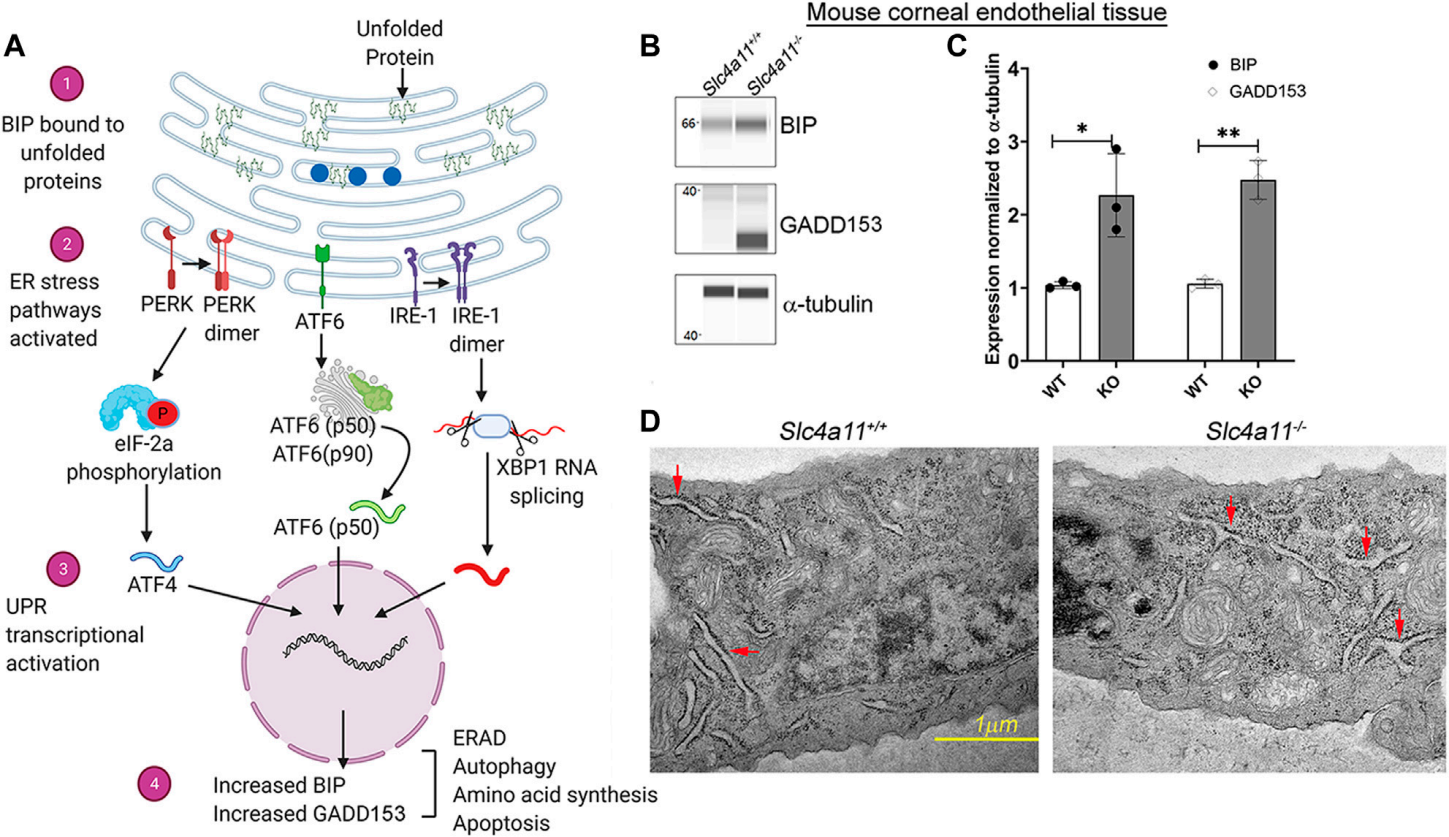
The ER Stress Response in WT and KO Mouse Corneal Endothelium. **(A)** ER stress through the unfolded protein response pathway is highly regulated through three main signaling pathways—ATF6, IRE1, and PERK. In a normal cell, the protein BIP (blue circles) is associated with the above mentioned molecules, thereby preventing the activation of ER stress associated signal transductions. With the accumulation of unfolded proteins the chaperone BIP associates with them (1) and less with the signaling molecules thereby activating the signaling pathways (2) and the ER stress associated transcriptional machinery (3). Increased GADD153 and BIP levels are considered to be two of the major outcomes of ER stress pathways (4). **(B)** Wes immunoassay analysis to determine the expression of ER stress markers, BIP and GADD153 in *Slc4a11* WT and KO corneal endothelial tissue. **(C)** Quantification of Wes immunoblots from Panel **(B)** n = 3, **p < 0.001 **(D)** Electron micrograph of *Slc4a11* ^+/+^ and *Slc4a11* ^−/−^ corneal endothelial tissue. Red arrows point to the ER. Scale—1 μM.

ER is the major site for protein folding in eukaryotes. When the protein folding machinery is overwhelmed by the influx of nascent polypeptides, ER stress ensues. The Unfolded Protein Response (UPR) pathway is activated during ER stress to promote cell survival through three distinct pathways (Figure 1A). However, chronic UPR activation leads to ER-stress-induced apoptosis (Adams et al., 2019; Bartoszewska and Collawn, 2020).

Misfolded proteins trigger the unfolded protein response (UPR) through three primary sensors, Inositol-Requiring Enzyme 1 α (IRE1-α), Protein kinase R-like Endoplasmic Reticulum Kinase (PERK), and Activating transcription factor 6 (ATF6). Activation of UPR leads to increased expression of ER chaperone protein BIP (Binding Immunoglobulin Protein) and chronic ER stress induces apoptosis through ER-related apoptosis protein GADD153 (Growth Arrest DNA Damage protein153, also known as CHOP) (Figure 1A) (Qi et al., 2017). In the mouse corneal endothelium, we observed increased BIP and GADD153 expression in 10 week old *Slc4a11* ^−/−^ animals but not in the age-matched *Slc4a11* ^+/+^ animals (Figures 1B,C). Another established characteristic of ER stress is the presence of dilated ER lumen (Bernales et al., 2006; Despa, 2009; Hartley et al., 2010; Schönthal, 2012). Therefore, we conducted electron microscopy to determine whether there are any structural changes in the ER of the corneal endothelia of *Slc4a11* ^−/−^ mice. Ten-week old *Slc4a11* ^−/−^ mice corneal endothelia reveal the presence of dilated ER lumen that was not observed in WT samples of age-matched animals (Figure 1D).

### Glutamine Induced Activation of UPR in *Slc4a11* ^*−/−*^ Corneal Endothelial Cells

**FIGURE 2:**
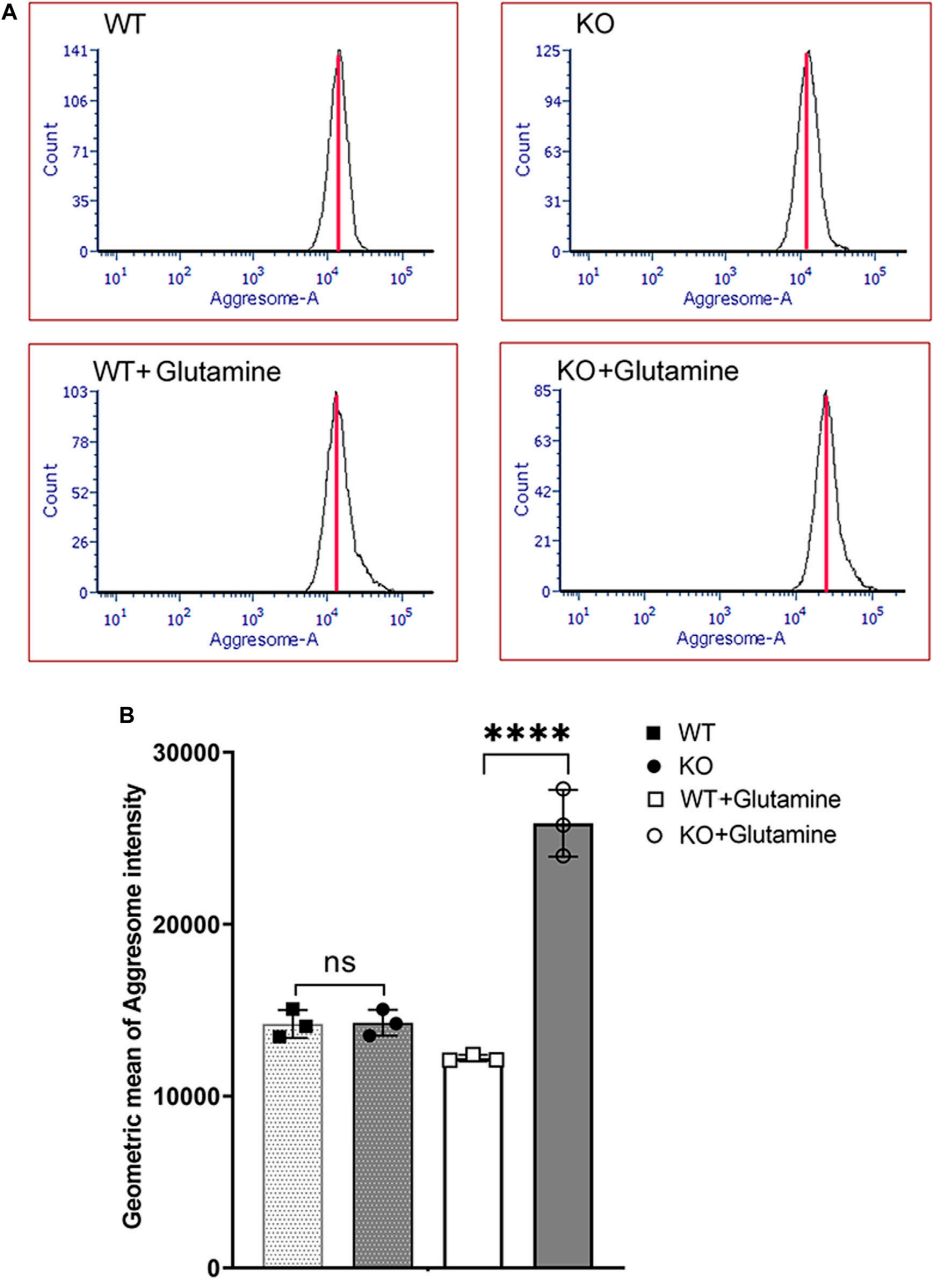
Aggresome Formation in Immortalized WT and *Slc4a11* KO MCEC. **(A)** Flow cytometry analysis of *Slc4a11* WT and KO MCEC to determine the presence of aggresomes (marker for unfolded proteins in the ER). **(B)** Quantification of the Geometric mean of Aggresome Intensity. Students t-test. n = 3, ****p < 0.0001.

Glutamine-induced mitochondrial ROS is the primary cellular stress in *Slc4a11* ^*−/−*^ corneal endothelium (Ogando et al., 2019). To determine its contribution toward ER stress, we treated *Slc4a11* ^*+/+*^ and *Slc4a11* ^*−/−*^ immortalized corneal endothelial cells with or without glutamine. Flow cytometry analysis showed a significant increase in misfolded protein aggregates (aggresomes) in *Slc4a11* ^*−/−*^ compared to WT cells in glutamine media (Figures 2A,B). Unstained cells were used as negative control, and cells treated with the ER stress inducer Thapsigargin, was used as positive control for aggresome formation (Supplementary Figure S1).

**FIGURE 3:**
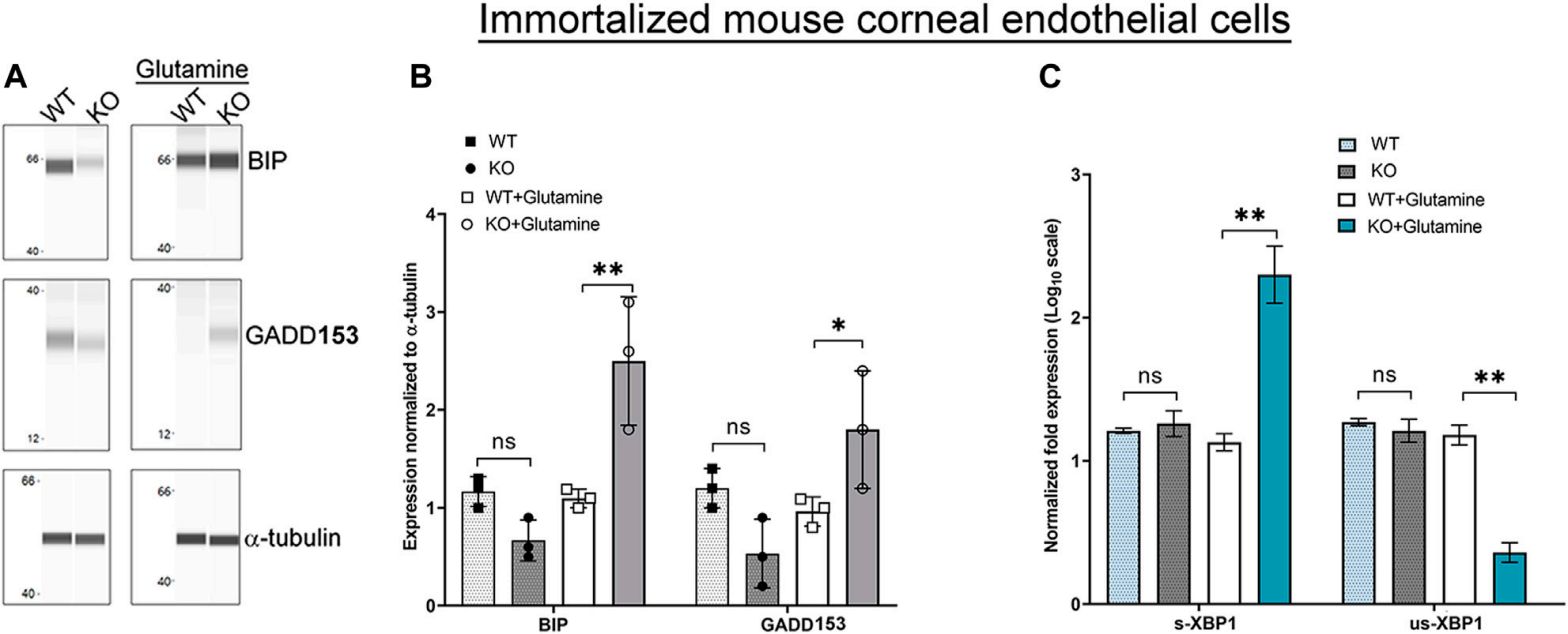
ER Stress Response in Immortalized WT and *Slc4a11* KO MCEC. **(A)** Wes analysis showing the expression of ER stress markers, BIP and GADD153 in *Slc4a11* WT and KO corneal endothelial cells treated in media with or without glutamine. α-tubulin was used as a loading control. **(B)** Quantification of Wes immunoassay. Student’s t-test. n = 3, **p < 0.001, *p < 0.0 **(C)** Q-PCR results showing the transcript levels of spliced-XBP1 (s-XBP1), and unspliced-XBP1 (us-XBP1) levels in *Slc4a11* WT and KO MCEC. Student’s t-test. n = 3, **p < 0.001.

Glutamine incubation also elevated expression of UPR associated proteins, BIP and GADD153 in *Slc4a11* ^*−/−*^ cells consistent with the accumulation of misfolded proteins as an outcome of ER stress (Figures 3A,B). Splicing of X-Box Binding Protein-1 (XBP1) mRNA occurs in response to the IRE-1α pathway (Figure 1A). Therefore, if there is ER stress and IRE-1α is activated we expect an increase in the levels of spliced XBP1, but not the unspliced version. Using primers (Table 1) that can bind to unspliced XBP1, and spliced XBP1 (Yoon et al., 2019), we conducted real-time PCR. *Slc4a11* ^*−/−*^ cells in the presence of glutamine had increased levels of the spliced XBP1 transcripts (Figure 3C), whereas the unspliced XBP1 levels were decreased. This result is consistent with ER stress induced activation of IRE-1α pathway.

### Glutamine Decreased ER [Ca^2+^] Release in *Slc4a11* ^*−/−*^ Corneal Endothelial Cells

**FIGURE 4:**
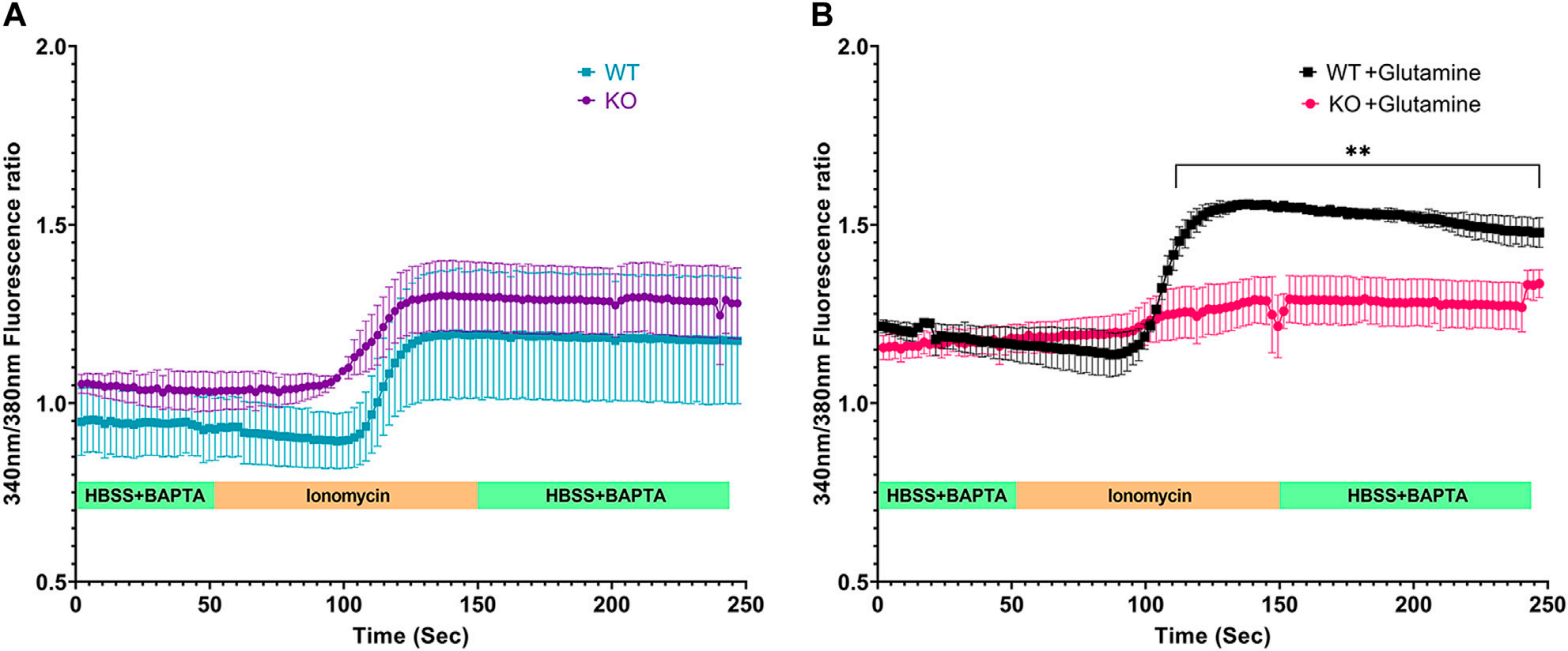
Calcium efflux from internal stores in Immortalized WT and *Slc4a11* KO MCEC. **(A)** *Slc4a11* WT and KO cells were cultured in media without glutamine, loaded with Fura-2 and perfused with Hanks Buffer with BAPTA to establish a baseline (0–50 s), followed by perfusion with ionomycin for 100 s **(B)** *Slc4a11* WT and KO cells were cultured in media with glutamine. The cells were perfused with Hanks Buffer with BAPTA to establish a baseline (0–50 s), followed by perfusion with ionomycin for 100 s. Student’s t-test. n = 3, **p < 0.01.

ER is the largest reservoir of Ca^2+^ in eukaryotic cells. We used the calcium indicator dye, Fura-2, to measure cytosol [Ca^2+^]. A baseline fluorescence ratio was established using Hanks Balanced Saline Solution (HBSS) containing calcium chelator BAPTA so that extracellular calcium was minimal. Following this, the cells were perfused with the calcium ionophore ionomycin, which under these conditions releases Ca^2+^ from ER intracellular stores. Live-cell microscopy revealed similar baseline and ionomycin stimulated change in cytosolic [Ca^2+^] for both *Slc4a11* ^*+/+*^ and *Slc4a11* ^*−/−*^ cells when perfused without glutamine (Figure 4A). However in the presence of glutamine, ionomycin released significantly less calcium into the cytosol of *Slc4a11* ^*−/−*^ cells indicating that the ER calcium store is deficient (Figure 4B).

### Mitochondrial ROS Increases ER Stress in Wild-type Corneal Endothelial Cells

**FIGURE 5:**
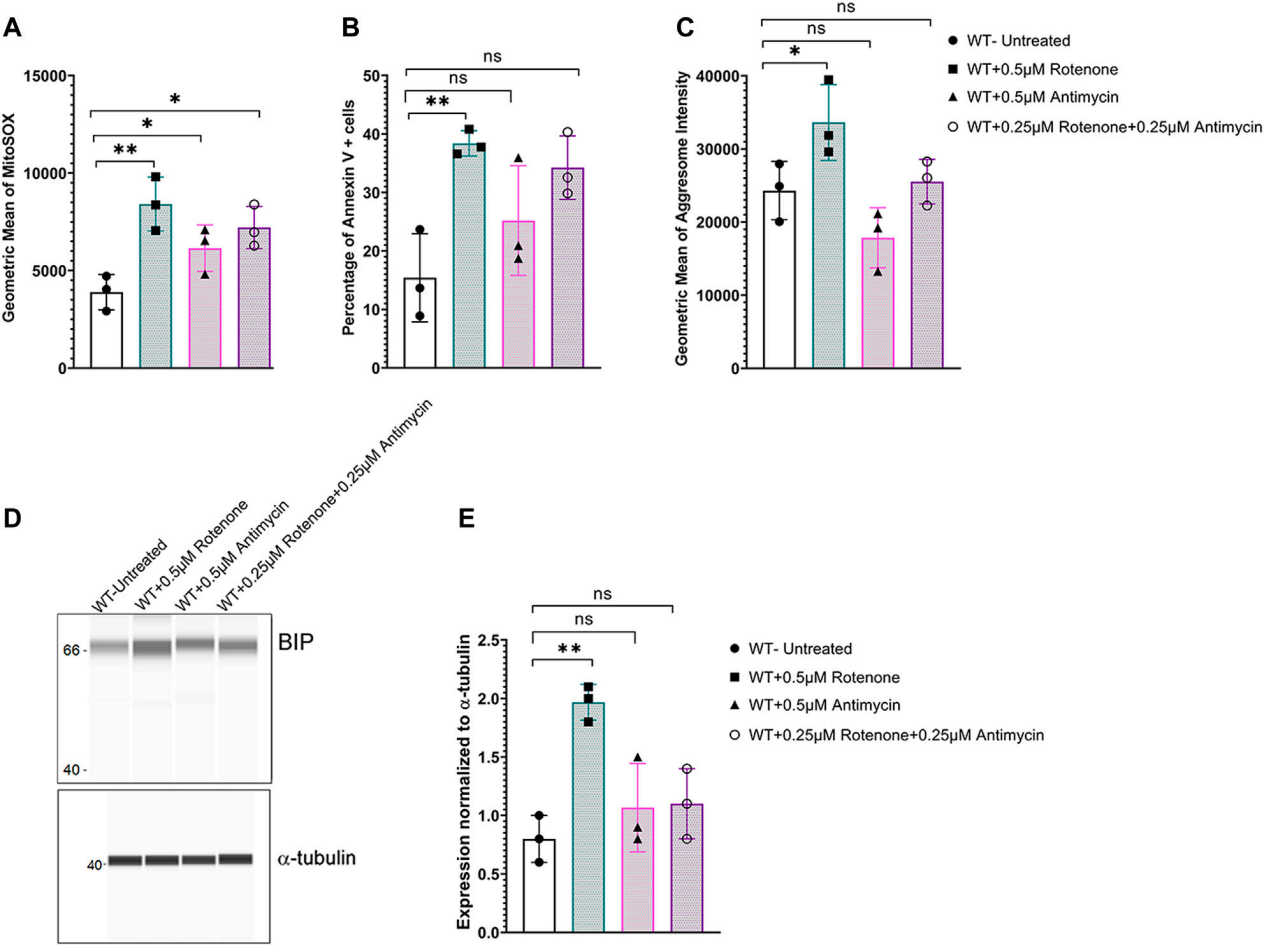
Mitochondrial ROS induces ER stress in *Slc4a11* WT MCEC. Cells were treated with 0.5 μM Antimycin, 0.25 μM each of rotenone and antimycin, or 0.5 μM Rotenone for 18 h. **(A)** Flow cytometry analysis of mitochondrial oxidative stress using MitoSox staining. N = 3. *p < 0.05, **p < 0.01. **(B)** Flow cytometry analysis of apoptotic cells using Annexin V staining. N = 3, **p < 0.01, ns-not significant. **(C)** Flow cytometry analysis to detect the presence of aggresomes. N = 3, *p < 0.05, ns-not significant. **(D,E)** Wes immunoassay for BIP and quantification. N = 3, *p < 0.05, ns-not significant.

To determine if oxidative stress was sufficient to induce ER stress, WT corneal endothelial cells were treated with mitochondrial ROS inducers - rotenone (mitochondrial electron transport chain complex 1 inhibitor) or antimycin A (mitochondrial electron transport chain complex III inhibitor) or a combination of both drugs. Flow cytometry analysis of MitoSOX indicated that all three treatment conditions increased mitochondrial oxidative stress (Figure 5A), while significant apoptotic cell death was evident only with 0.5 μM rotenone treatment (Figure 5B). Significant increase in aggresome levels (Figure 5C) as well as elevation in ER stress marker, BIP (Figures 5D,E), were observed with 0.5 μM Rotenone treatment indicating that mitochondrial oxidative stress alone is sufficient to cause ER stress and that it is not simply due to the lack of Slc4a11.

### MitoQ Reduced ER Stress and Improved ER Ca^2+^ Release in *Slc4a11* ^*−/−*^ Cells

**FIGURE 6:**
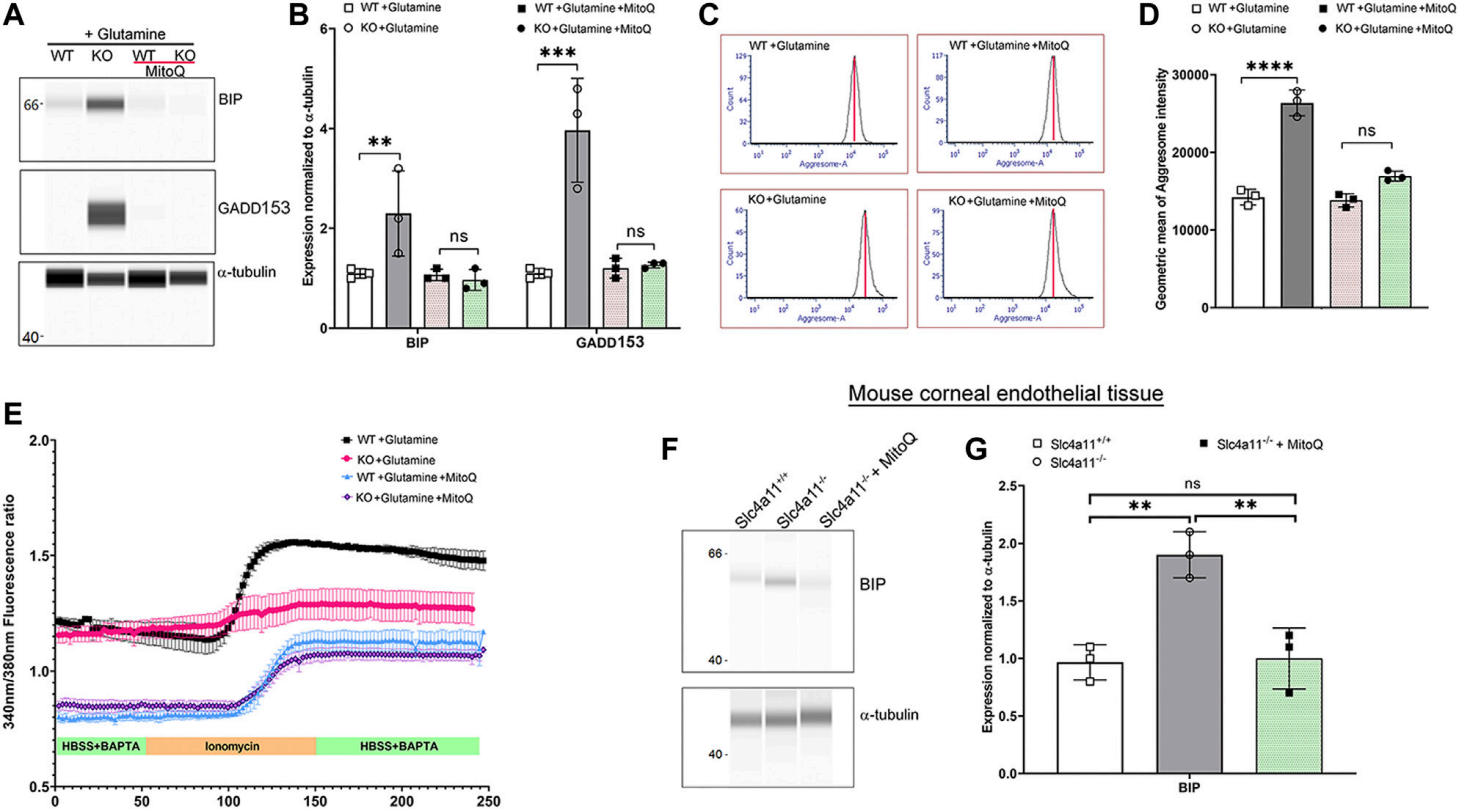
Quenching Mito ROS reverses ER stress in *Slc4a11* KO. **(A)** Wes immunoassay for BIP and GADD153 of *Slc4a11* WT and KO MCEC treated with 2 μM MitoQ. **(B)** Quantification of Wes immunoassay. N = 3. **p < 0.01, ***p < 0.001. Student’s t-test. **(C)** Flow cytometry analysis of WT and KO MCEC treated with 2 μM MitoQ to determine the presence of aggresomes. **(D)** Quantification of the geometric mean of aggresome intensity. N = 3. *p* < 0.0001. Student’s t-test. **(E)** *Slc4a11* WT and KO MCEC were cultured in media with glutamine ± 2 μM MitoQ. The cells were loaded with Fura-2 and perfused with Hanks Buffer with BAPTA to establish a baseline (0–50 s), followed by perfusion with ionomycin for 100 s. **(F)** Wes immunoassay for BIP in *Slc4a11* WT and KO corneal endothelial tissue of animals treated with or without MitoQ. **(G)** Quantification of Wes immunoassay. N = 3. **p < 0.01, ns-not significant.

In the absence of the ammonia sensitive mitochondrial uncoupling by Slc4a11, glutamine catabolism leads to mitochondrial membrane hyperpolarization that elevates mitochondrial ROS in corneal endothelial cells (Ogando et al., 2019). Since we now show that glutamine is causing ER stress, we asked whether quenching mitochondrial ROS can decrease ER stress in *Slc4a11* ^*−/−*^ cells. *Slc4a11* ^−/−^ corneal endothelial cells were incubated with 2 μM MitoQ for 24 h in assay media, which was previously shown to significantly reduce mitochondrial ROS (Shyam et al., 2021). Figures 6A,B show that this treatment significantly reduced BIP and GADD153 expression, and aggresome levels in KO cells (Figures 6C,D). Interestingly, Figure 6E shows that MitoQ in the presence of glutamine lowered baseline calcium in both WT and KO corneal endothelial cells and restored the release of calcium from internal stores by ionomycin in KO cells to a level similar to that observed in glucose alone (see Figure 4A).

To determine whether MitoQ was sufficient to improve ER stress *in vivo*, 8-week old *Slc4a11* ^−/−^ animals were injected with this drug on alternate days over the course of 4 weeks (Shyam et al., 2021). Following MitoQ treatment, BIP expression in dissected corneal endothelium was determined using Wes immunoassay. Significant reduction in BIP levels were observed in *Slc4a11* ^−/−^ animals compared to littermate controls when treated with MitoQ (Figures 6F,G). These observations are consistent with our previous findings in which MitoQ treatment alleviated autophagy and lysosomal dysfunction in *Slc4a11* ^−/−^ animals (Shyam et al., 2021).

## Discussion

In addition to glucose, glutamine has been established as an important metabolite used by corneal endothelium (Zhang et al., 2017a; Zhang et al., 2017b; Ogando et al., 2019; Hamuro et al., 2020; Jin et al., 2020; Ogando and Bonanno, 2022). Moreover, the high expression of *Slc4a11* in mitochondria of corneal endothelial cells reduces glutamine-induced generation of superoxide and oxidative damage (Ogando et al., 2019). In the current study, we show that glutamine (*in vivo* or *in vitro*) in the absence of *Slc4a11* leads to ER stress that can be reversed by quenching mitochondrial ROS. Mitochondrial ROS also triggers lysosomal dysfunction with autophagy impairment in *Slc4a11* ^*−/−*^ corneal endothelial cells (Shyam et al., 2021). Since lysosomes are derived from ER-Golgi, our findings suggest that ROS induced ER stress is an important trigger for cellular dysfunction in the *Slc4a11* KO model.

In many pathological conditions, ER stress manifests when the capacity of this organelle to fold proteins becomes saturated, thereby leading to the accumulation of misfolded proteins (Ren et al., 2021). Evolutionarily conserved unfolded protein response acts as an ER to nucleus signal to trigger signaling pathways that can circumvent cell death. Even though proteasomal degradation of unfolded proteins is the canonical ER-associated clearance mechanism (Ren et al., 2021), recent evidence indicates that ER stress can activate autophagy as a cell survival mechanism (Bernales et al., 2006; Yorimitsu et al., 2006; Rashid et al., 2015). Cross-talk between ROS, ER stress and autophagy is well-documented in other cell types (Senft and Ronai, 2015) and it is possible that ROS-mediated ER stress may activate autophagy in CHED. However, ROS induced lysosomal dysfunction prevents the degradation of autophagosome contents (Shyam et al., 2021). Together, these studies indicate the effect of elevated mitochondrial ROS on multiple organelle functions in corneal endothelial cells and suggest that dysfunctional lysosomes may stem from ER stress.

ER is the major reservoir of Ca^2+^ in the cell. In the presence of glutamine, both *Slc4a11* ^*+/+*^ and *Slc4a11* ^−/−^ cells showed slightly elevated cytosolic [Ca^2+^]; however, we noticed a significant decrease in ionomycin induced calcium efflux from internal stores in *Slc4a11* ^−/−^ cells in glutamine. These results suggest that ER stress induced by glutamine in *Slc4a11* ^−/−^ cells causes reduced Ca^2+^ release either due to impaired ER Ca^2+^ loading and/or dysfunctional release mechanisms. That treatment with MitoQ, which is known to reduce ROS levels in *Slc4a11* ^−/−^ cells (Shyam et al., 2021) improved Ca^2+^ efflux from the internal stores, supports the notion that ER stress is caused by mitochondrial ROS production. Interestingly, glutamine significantly raised cytosolic Ca^2+^ in both WT and KO cells and MitoQ lowered cytosolic Ca^2+^ levels. This suggests that mitochondrial activity and ROS production (high in KO and lower in WT) influence mitochondrial and/or ER Ca^2+^ levels (Feno et al., 2019; Delierneux et al., 2020).

Elevated ROS (Guha et al., 2017; Ogando et al., 2019), dysfunctional autophagy (Shyam et al., 2021), and ER stress are characteristics of loss of *Slc4a11*. Similar to the CHED model, increased ROS (Ong Tone et al., 2020) and ER distress (Okumura et al., 2017a) are present in FECD. Whether ROS induces ER stress in FECD is not known. With the evidence of organelle crosstalk in corneal endothelial cells, it is plausible that ER stress may result from elevated ROS in FECD.

## Conclusion

**FIGURE 7:**
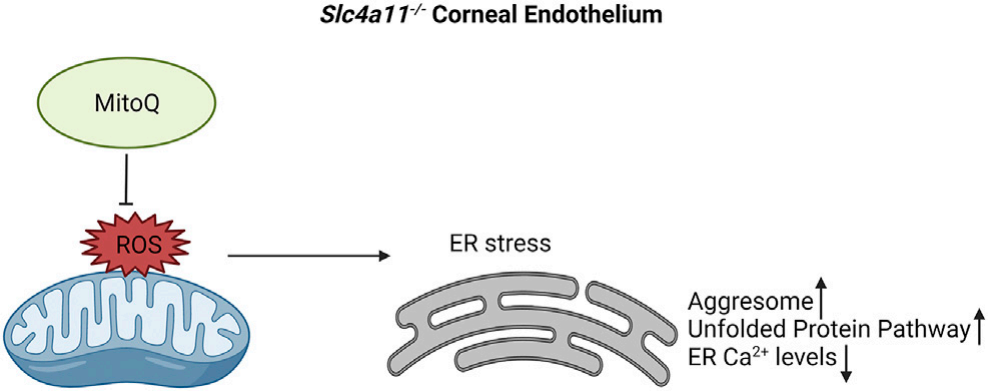
Proposed mechanism for ER stress in *Slc4a11* ^*−/−*^ corneal endothelium. Elevated mitochondrial ROS leads to ER stress. This results in decreased ER Ca2+ levels, elevated levels of aggresomes and increase in Unfolded Protein Pathway. Use of mitochondrial ROS quencher, MitoQ, alleviates ER stress in *Slc4a11* ^*−/−*^ corneal endothelium.

Mitochondrial ROS increases ER stress, decreases ER Ca^2+^ stores and upregulates the unfolded protein response pathway in *Slc4a11* ^−/−^ corneal endothelium. Our study is the first to determine the presence of ER stress in an animal model of CHED and provide evidence that this is attributable to mitochondrial ROS (Figure 7).

## Data Availability

The original contributions presented in the study are included in the article/Supplementary Material, further inquiries can be directed to the corresponding author.

## References

[B1] Adams C. J. Kopp M. C. Larburu N. Nowak P. R. Ali M. M. U. (2019). Structure and Molecular Mechanism of ER Stress Signaling by the Unfolded Protein Response Signal Activator IRE1. Front. Mol. Biosci. 6, 11. 10.3389/fmolb.2019.00011 30931312 PMC6423427

[B2] Aldave A. J. Han J. Frausto R. F. (2013). Genetics of the Corneal Endothelial Dystrophies: an Evidence-Based Review. Clin. Genet. 84, 109–119. 10.1111/cge.12191 23662738 PMC3885339

[B3] Aldave A. J. Yellore V. S. Bourla N. Momi R. S. Khan M. A. Salem A. K. (2007). Autosomal Recessive CHED Associated with Novel Compound Heterozygous Mutations in SLC4A11. Cornea 26, 896–900. 10.1097/ICO.0b013e318074bb01 17667634

[B4] Bartoszewska S. Collawn J. F. (2020). Unfolded Protein Response (UPR) Integrated Signaling Networks Determine Cell Fate during Hypoxia. Cell. Mol. Biol. Lett. 25, 18. 10.1186/s11658-020-00212-1 32190062 PMC7071609

[B5] Bernales S. McDonald K. L. Walter P. (2006). Autophagy Counterbalances Endoplasmic Reticulum Expansion during the Unfolded Protein Response. Plos Biol. 4, e423. 10.1371/journal.pbio.0040423 17132049 PMC1661684

[B6] Delierneux C. Kouba S. Shanmughapriya S. Potier-Cartereau M. Trebak M. Hempel N. (2020). Mitochondrial Calcium Regulation of Redox Signaling in Cancer. Cells 9, 432. 10.3390/cells9020432 PMC7072435 32059571

[B7] Despa F. (2009). Dilation of the Endoplasmic Reticulum in Beta Cells Due to Molecular Overcrowding? Biophysical Chem. 140, 115–121. 10.1016/j.bpc.2008.12.003 19121888

[B8] Feno S. Butera G. Vecellio Reane D. Rizzuto R. Raffaello A. (2019). Crosstalk between Calcium and ROS in Pathophysiological Conditions. Oxidative Med. Cell Longevity 2019, 1–18. 10.1155/2019/9324018 PMC6507098 31178978

[B9] Guha S. Chaurasia S. Ramachandran C. Roy S. (2017). SLC4A11 Depletion Impairs NRF2 Mediated Antioxidant Signaling and Increases Reactive Oxygen Species in Human Corneal Endothelial Cells during Oxidative Stress. Sci. Rep. 7, 4074. 10.1038/s41598-017-03654-4 28642546 PMC5481427

[B10] Hamuro J. Deguchi H. Fujita T. Ueda K. Tokuda Y. Hiramoto N. (2020). Polarized Expression of Ion Channels and Solute Carrier Family Transporters on Heterogeneous Cultured Human Corneal Endothelial Cells. Invest. Ophthalmol. Vis. Sci. 61, 47. 10.1167/iovs.61.5.47 PMC7405722 32455435

[B11] Han S. B. Ang H.-P. Poh R. Chaurasia S. S. Peh G. Liu J. (2013). Mice with a Targeted Disruption ofSlc4a11Model the Progressive Corneal Changes of Congenital Hereditary Endothelial Dystrophy. Invest. Ophthalmol. Vis. Sci. 54, 6179–6189. 10.1167/iovs.13-12089 23942972

[B12] Hartley T. Siva M. Lai E. Teodoro T. Zhang L. Volchuk A. (2010). Endoplasmic Reticulum Stress Response in an INS-1 Pancreatic β-cell Line with Inducible Expression of a Folding-Deficient Proinsulin. BMC Cel Biol 11, 59. 10.1186/1471-2121-11-59 PMC2921384 20659334

[B13] Jin M. Wang Y. Wang Y. Li Y. Wang G. Liu X. (2020). Protective Effects Oncorneal Endothelium during Intracameral Irrigation Using N-(2)-l-alanyl-l-Glutamine. Front. Pharmacol. 11, 369. 10.3389/fphar.2020.00369 32292346 PMC7118711

[B14] Jun A. S. Meng H. Ramanan N. Matthaei M. Chakravarti S. Bonshek R. (2012). An Alpha 2 Collagen VIII Transgenic Knock-In Mouse Model of Fuchs Endothelial Corneal Dystrophy Shows Early Endothelial Cell Unfolded Protein Response and Apoptosis. Hum. Mol. Genet. 21, 384–393. 10.1093/hmg/ddr473 22002996 PMC3276279

[B15] Langford M. P. Gosslee J. M. Liang C. Chen D. Redens T. B. Welbourne T. C. (2007). Apical Localization of Glutamate in GLAST-1, Glutamine Synthetase Positive Ciliary Body Nonpigmented Epithelial Cells. Clin. Ophthalmol. 1, 43–53. 19668465 PMC2699989

[B16] Loganathan S. K. Casey J. R. (2014). Corneal Dystrophy-Causing SLC4A11 Mutants: Suitability for Folding-Correction Therapy. Hum. Mutat. 35, 1082–1091. 10.1002/humu.22601 24916015

[B17] Ogando D. G. Bonanno J. A. (2022). RNA Sequencing Uncovers Alterations in Corneal Endothelial Metabolism, Pump and Barrier Functions of Slc4a11 KO Mice. Exp. Eye Res. 214, 108884. 10.1016/j.exer.2021.108884 34871568 PMC8792362

[B18] Ogando D. G. Choi M. Shyam R. Li S. Bonanno J. A. (2019). Ammonia Sensitive SLC4A11 Mitochondrial Uncoupling Reduces Glutamine Induced Oxidative Stress. Redox Biol. 26, 101260. 10.1016/j.redox.2019.101260 31254733 PMC6604051

[B19] Ogando D. G. Shyam R. Kim E. T. Wang Y.-C. Liu C.-Y. Bonanno J. A. (2021). Inducible Slc4a11 Knockout Triggers Corneal Edema through Perturbation of Corneal Endothelial Pump. Invest. Ophthalmol. Vis. Sci. 62, 28. 10.1167/iovs.62.7.28 PMC8826551 34190974

[B20] Okumura N. Hashimoto K. Kitahara M. Okuda H. Ueda E. Watanabe K. (2017a). Activation of TGF-β Signaling Induces Cell Death via the Unfolded Protein Response in Fuchs Endothelial Corneal Dystrophy. Sci. Rep. 7, 6801. 10.1038/s41598-017-06924-3 28754918 PMC5533742

[B21] Okumura N. Kitahara M. Okuda H. Hashimoto K. Ueda E. Nakahara M. (2017b). Sustained Activation of the Unfolded Protein Response Induces Cell Death in Fuchs' Endothelial Corneal Dystrophy. Invest. Ophthalmol. Vis. Sci. 58, 3697. 10.1167/iovs.16-21023 28727885

[B22] Ong Tone S. Kocaba V. Böhm M. Wylegala A. White T. L. Jurkunas U. V. (2021). Fuchs Endothelial Corneal Dystrophy: The Vicious Cycle of Fuchs Pathogenesis. Prog. Retin. Eye Res. 80, 100863. 10.1016/j.preteyeres.2020.100863 32438095 PMC7648733

[B23] Qi L. Tsai B. Arvan P. (2017). New Insights into the Physiological Role of Endoplasmic Reticulum-Associated Degradation. Trends Cel Biol. 27, 430–440. 10.1016/j.tcb.2016.12.002 PMC5440201 28131647

[B24] Rashid H.-O. Yadav R. K. Kim H.-R. Chae H.-J. (2015). ER Stress: Autophagy Induction, Inhibition and Selection. Autophagy 11, 1956–1977. 10.1080/15548627.2015.1091141 26389781 PMC4824587

[B25] Ren H. Zhai W. Lu X. Wang G. (2021). The Cross-Links of Endoplasmic Reticulum Stress, Autophagy, and Neurodegeneration in Parkinson's Disease. Front. Aging Neurosci. 13, 691881. 10.3389/fnagi.2021.691881 34168552 PMC8218021

[B26] Schönthal A. H. (2012). Endoplasmic Reticulum Stress: Its Role in Disease and Novel Prospects for Therapy. Scientifica 2012, 1–26. 10.6064/2012/857516 PMC3820435 24278747

[B27] Senft D. Ronai Z. e. A. (2015). UPR, Autophagy, and Mitochondria Crosstalk Underlies the ER Stress Response. Trends Biochem. Sci. 40, 141–148. 10.1016/j.tibs.2015.01.002 25656104 PMC4340752

[B28] Shyam R. Ogando D. G. Choi M. Liton P. B. Bonanno J. A. (2021). Mitochondrial ROS Induced Lysosomal Dysfunction and Autophagy Impairment in an Animal Model of Congenital Hereditary Endothelial Dystrophy. Invest. Ophthalmol. Vis. Sci. 62, 15. 10.1167/iovs.62.12.15 PMC8458782 34533563

[B29] Shyam R. Ogando D. G. Kim E. T. Murugan S. Choi M. Bonanno J. A. (2022). Rescue of the Congenital Hereditary Endothelial Dystrophy Mouse Model by Adeno-Associated Virus-Mediated Slc4a11 Replacement. Ophthalmol. Sci. 2, 100084. 10.1016/j.xops.2021.100084 PMC9432820 36051248

[B30] Vithana E. N. Morgan P. Sundaresan P. Ebenezer N. D. Tan D. T. H. Mohamed M. D. (2006). Mutations in Sodium-Borate Cotransporter SLC4A11 Cause Recessive Congenital Hereditary Endothelial Dystrophy (CHED2). Nat. Genet. 38, 755–757. 10.1038/ng1824 16767101

[B31] Xu J. Zhou Q. Xu W. Cai L. (2012). Endoplasmic Reticulum Stress and Diabetic Cardiomyopathy. Exp. Diabetes Res. 2012, 1–12. 10.1155/2012/827971 PMC3226330 22144992

[B32] Yoon S.-B. Park Y.-H. Choi S.-A. Yang H.-J. Jeong P.-S. Cha J.-J. (2019). Real-time PCR Quantification of Spliced X-Box Binding Protein 1 (XBP1) Using a Universal Primer Method. PLoS One 14, e0219978. 10.1371/journal.pone.0219978 31329612 PMC6645673

[B33] Yorimitsu T. Nair U. Yang Z. Klionsky D. J. (2006). Endoplasmic Reticulum Stress Triggers Autophagy. J. Biol. Chem. 281, 30299–30304. 10.1074/jbc.M607007200 16901900 PMC1828866

[B34] Zhang W. Li H. Ogando D. G. Li S. Feng M. Price F. W. (2017a). Glutaminolysis Is Essential for Energy Production and Ion Transport in Human Corneal Endothelium. EBioMedicine 16, 292–301. 10.1016/j.ebiom.2017.01.004 28117276 PMC5474426

[B35] Zhang W. Ogando D. G. Kim E. T. Choi M.-J. Li H. Tenessen J. M. (2017b). Conditionally Immortal Slc4a11−/− Mouse Corneal Endothelial Cell Line Recapitulates Disrupted Glutaminolysis Seen in Slc4a11−/− Mouse Model. Invest. Ophthalmol. Vis. Sci. 58, 3723–3731. 10.1167/iovs.17-21781 28738416 PMC5525555

