# Supplementary material for: Mitochondrial ROS in Slc4a11 KO Corneal Endothelial Cells Lead to ER Stress
Source: Front Cell Dev Biol. 2022 Apr 26;10:878395. doi: 10.3389/fcell.2022.878395 (PMC9086159; doi:10.3389/fcell.2022.878395)
Supplement: Supplementary file 1 [file Image1.pdf]

Supplementary Figure 1

A.

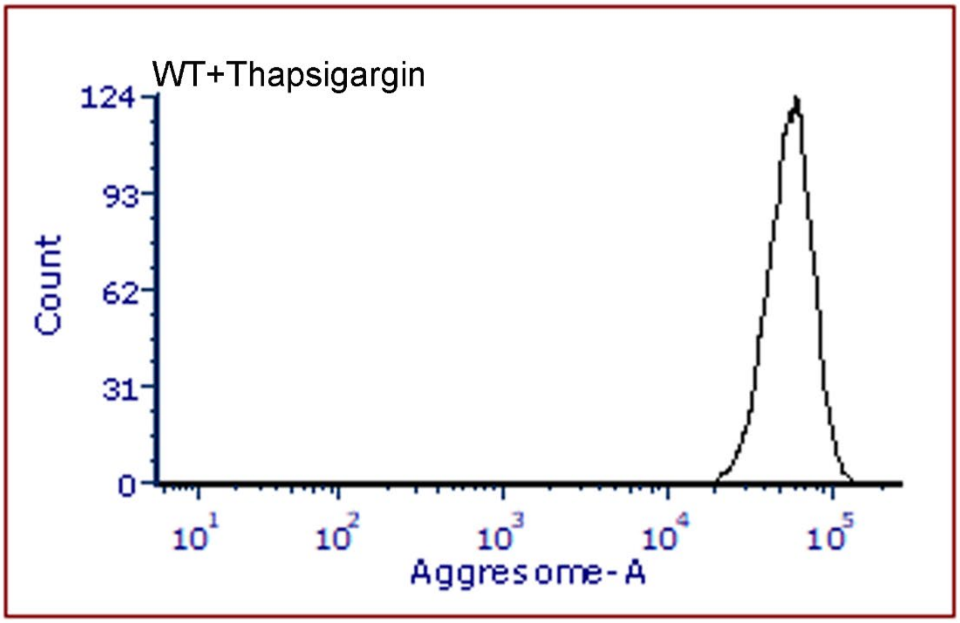

B.

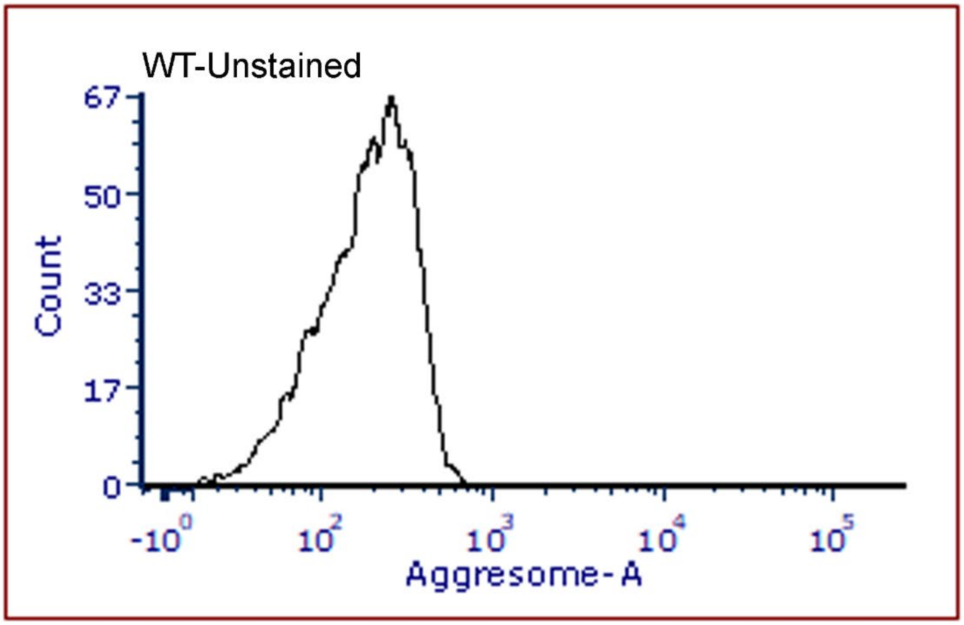

Fig. S1 – Histograms showing geometric mean of Aggresome intensity of WT cells treated with ER stress inducer, Thapsigargin and unstained WT cells.
